# Interplay between Cell Migration and Neurite Outgrowth Determines SH2B1β-Enhanced Neurite Regeneration of Differentiated PC12 Cells

**DOI:** 10.1371/journal.pone.0034999

**Published:** 2012-04-23

**Authors:** Chia-Ling Wu, Yu-Han Chou, Yu-Jung Chang, Nan-Yuan Teng, Hsin-Ling Hsu, Linyi Chen

**Affiliations:** 1 Institute of Molecular Medicine, National Tsing Hua University, Hsinchu, Taiwan, Republic of China; 2 Department of Medical Science, National Tsing Hua University, Hsinchu, Taiwan, Republic of China; 3 Department of Life Science, National Tsing Hua University, Hsinchu, Taiwan, Republic of China; 4 Division of Molecular and Genomic Medicine, National Health Research Institutes, Miaoli County, Taiwan, Republic of China; Hungarian Academy of Sciences, Hungary

## Abstract

The regulation of neurite outgrowth is crucial in developing strategies to promote neurite regeneration after nerve injury and in degenerative diseases. In this study, we demonstrate that overexpression of an adaptor/scaffolding protein SH2B1β promotes neurite re-growth of differentiated PC12 cells, an established neuronal model, using wound healing (scraping) assays. Cell migration and the subsequent remodeling are crucial determinants during neurite regeneration. We provide evidence suggesting that overexpressing SH2B1β enhances protein kinase C (PKC)-dependent cell migration and phosphatidylinositol 3-kinase (PI3K)-AKT-, mitogen activated protein kinase (MAPK)/extracellular signal-regulated protein kinase (ERK) kinase (MEK)-ERK-dependent neurite re-growth. Our results further reveal a cross-talk between pathways involving PKC and ERK1/2 in regulating neurite re-growth and cell migration. We conclude that temporal regulation of cell migration and neurite outgrowth by SH2B1β contributes to the enhanced regeneration of differentiated PC12 cells.

## Introduction

Neuronal injury and degeneration are responsible for various neurological diseases. The limited regeneration capacity restricts the recovery of neuronal damage. Thus, better understanding of the mechanisms for neuronal repair will facilitate clinical application of therapy toward neurological disorders. Peripheral nerve transection (axotomy) is often used as a neuronal injury model. During regeneration of the peripheral nervous system (PNS), cell body of the neurons must to receive appropriate signals to sustain intrinsic growth to ensure successful regeneration. Thus, the regulation of signaling cascades and downstream gene expression often determines the regeneration outcome [1], [2], [3]. For instance, axonal injury induces local activation and retrograde transport of extracellular signal-regulated protein kinase (ERK) [4], [5], [6] and c-Jun N-terminal kinase (JNK) [7], [8]. A study showed that mitogen activated protein kinase (MAPK)/ERK kinase (MEK) kinase 1 (MEKK1) controls neurite re-growth by balancing ERK1/2 and JNK2 signaling after experimental injury [9]. These studies suggest that activation of JNK and ERK and their interaction with the dynein/dynactin retrograde molecular motors is required for regeneration [1], [5], [7]. Moreover, overexpression of constitutively activated AKT has been shown to protect motor neurons from injury-induced cell death and thus promotes axonal regeneration [10], [11]. By intraperitoneally administrating vanadium compounds to stimulate the activation of phosphatidylinositol 3-kinase (PI3K)-AKT and MEK-ERK1/2 pathways, neurogenesis as well as newborn cells are increased in response to brain ischemia [12].

Neurotrophic factors, including nerve growth factor (NGF), fibroblast growth factor (FGF), glial cell -derived neurotrophic factor (GDNF), brain-derived neurotrophic factor (BDNF), neurotropin-3 (NT-3) and neurotropin-4/5 (NT-4/5), not only regulate neuronal development, but also play positive roles in enhancing regeneration [13], [14]. Evidence demonstrates that NGF promotes long distance axonal regeneration in cerulospinal axons and primary sensory axons [15], [16], [17]. As NGF binds to its receptor TrkA, trans-phosphorylation of the receptors leads to their activation. The phosphorylated tyrosine residues can serve as docking sites for signaling molecules within MEK-ERK, PI3K-AKT, and phospholipase Cγ (PLCγ)-Protein kinase C (PKC) pathways, to further transmit signals to downstream effectors [18]. Previous studies show that MEK-ERK pathway is essential for NGF-induced neurite outgrowth in pheochromocytoma-derived PC12 cell, an established neuronal model cell line [19], [20], [21]. Activation of PI3K-AKT, on the other hand, is required for the protection of PC12 cells from apoptosis as well as for the neuritogenesis of dorsal root ganglion (DRG) sensory neurons [22], [23], [24]. Interestingly, both Ras-Raf-ERK and PI3K-AKT pathways have been shown essential for NGF-induced axonal growth of embryonic DRG neurons [25]. Ras-Raf-ERK cascade regulates the axon elongation whereas PI3K-AKT signaling increases the axon caliber and branch [25]. These studies implicate the importance of ERK1/2, JNK, and PI3K-AKT pathways in neurite outgrowth.

**Figure 1:**
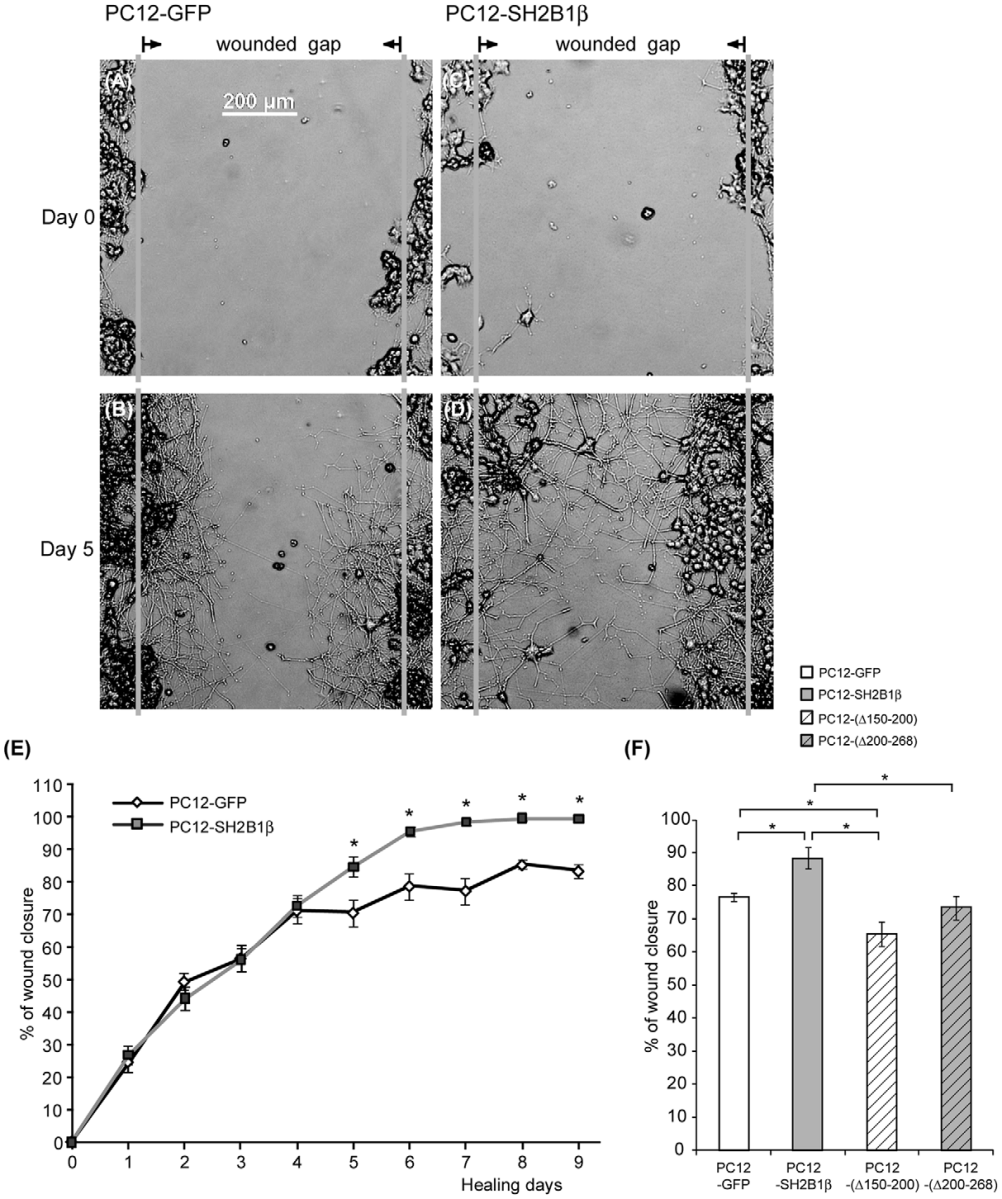
Overexpression of SH2B1β enhances wound healing of differentiated PC12 cells. PC12-GFP, PC12-SH2B1β cells (A–D) and PC12-GFP, PC12-SH2B1β, PC12-(Δ150–200), PC12-(Δ200–268) cells (F) were differentiated by 50 ng/ml NGF treatment for 8 days. On day 8, differentiated cells were scraped by P10 tips and defined as healing day 0. Live cell images of the same wounded gaps were taken every 24 h for at least 7 days. Representative images are shown. Scale bar: 200 μm. (E) The percentages of wound closure were quantified as described in the Materials and Methods. Values are mean ± S.E.M. from seven independent experiments for healing days 0–7 and four independent experiments for healing days 8 and 9. (F) The percentages of wound closure on healing day 6 are show. Data are mean ± S.E.M. from three independent experiments. (*: compared to the percentages of PC12-GFP cells on the same day, P<0.05, paired Student's t-test).

Cell migration is instrumental for injury-induced neurogenesis and tissue regeneration [26], [27], [28], [29], [30]. It has been shown that progenitor cells from periventricular region proliferate and migrate into the hippocampus to regenerate new neurons after ischemia, thus reduce neurological deficits *in vivo* [31]. Evidence suggests that activities of PLC and PKC, and intracellular Ca^2+^ levels are important regulators of cell migration in the developing brain [32]. PLC is the key enzyme which mediates the balance between intracellular Ca^2+^ and cellular phosphoinositide. PLC isozymes mainly localize in cytosol, and translocate to plasma membrane, where PLC hydrolyzes phosphatidylinositol 4,5-bisphosphate (PIP2) into inositol 1,4,5-trisphosphate (IP3) and diacylglycerol (DAG), as stimulated by hormones or neurotransmitters [33]. The produced second messenger IP3 increases endoplasmic reticulum-released Ca^2+^ and DAG regulates the activation of PKC. One of the PLC isozymes mainly regulated by receptor and cytosolic tyrosine kinase is PLCγ1 [34], [35], which expresses in lung, thymus, and highly expresses in neurons of adult rat brain [36]. In addition to growth factors-mediated chemotaxis, a study indicates that PLCγ1 is an essential molecule for cell motility in integrin signaling [37], suggesting the general role of PLCγ1 in cell motility. PKC isoforms are known to influence cell morphogenesis by remodeling microfilaments [38]. They have also been implicated in diverse signal propagation [39]. The ability of neurons to respond to injury, integrate signals, and remodel actin cytoskeleton determines the recovery outcome. Increasing evidence suggests that protein scaffolds are involved in temporally and spatially organizing signaling events to downstream transcriptional activity and actin remodeling.

One such scaffolding protein, SH2B1, belongs to the SH2B adaptor protein family [40], [41]. SH2B1β (β splice variant) has been shown to bind to multiple receptor tyrosine kinases, including receptors for NGF, FGF, and GDNF [42], [43], [44], [45], [46], [47]. SH2B1 is necessary for survival of sympathetic neurons [45]. It positively regulates the Akt/Forkhead pathway and has recently been shown to reduce oxidative stress-induced cell death in PC12 cells, hippocampal neurons and *Drosophila* [22], [48], [49]. Overexpressing SH2B1β also enhances both NGF-, FGF1- and GDNF-induced neuronal differentiation of PC12 cells [42], [43], [44], [45]. Given its ability to promote neurite outgrowth and survival, we hypothesize that SH2B1β may play a positive role in neuronal regeneration. This study investigated the role of SH2B1β in the regeneration of injured differentiated PC12 cells.

## Materials and Methods

### Reagents

Anti-ERK1/2 antibody and phorbol 12-myristate 13 acetate (PMA or TPA) were obtained from Sigma (S. Louis, MO). Anti-pPLCγ1(Tyr783), pSrc(Tyr416) family antibodies and horseradish peroxidase (HRP)-conjugated goat anti-mouse IgG were obtained from Cell Signaling (Danvers, MA). Anti-pERK1/2(Thr202, Tyr204) was purchased from Bioworld (Minneapolis, MN). Anti-Histone deacetylases (HDAC) and PLCγ1 antibodies were obtained from Millipore (Billerica, MA). Neuronal class III β-tubulin (Tuj1) was obtained from Covance (Richmond, CA). Alexa-Flour 555-conjugated goat anti-mouse antibody was obtained from Invitrogen (Carlsbad, CA). NGF and rat-tail collagen I were purchased from BD Bioscience (Bedford, MA). Protein Assay Kit (PAK500) was purchased from Strong Biotech Corporation, Taiwan. BCA assay reagent was purchased from SantaCruz Biotechnology (Santa Cruz, CA). IRDye800CW-labeled anti-rabbit secondary antibody was purchased from LI-COR Biosciences (Lincoln, NE). CF^TM^680 goat anti-mouse IgG was purchased from Biotium, Inc (Hayward, CA). U0126, LY294002 and Bisindolyleimide I hydrochloride (Bis) were purchased from Calbiochem (San Diego, CA).

**Figure 2:**
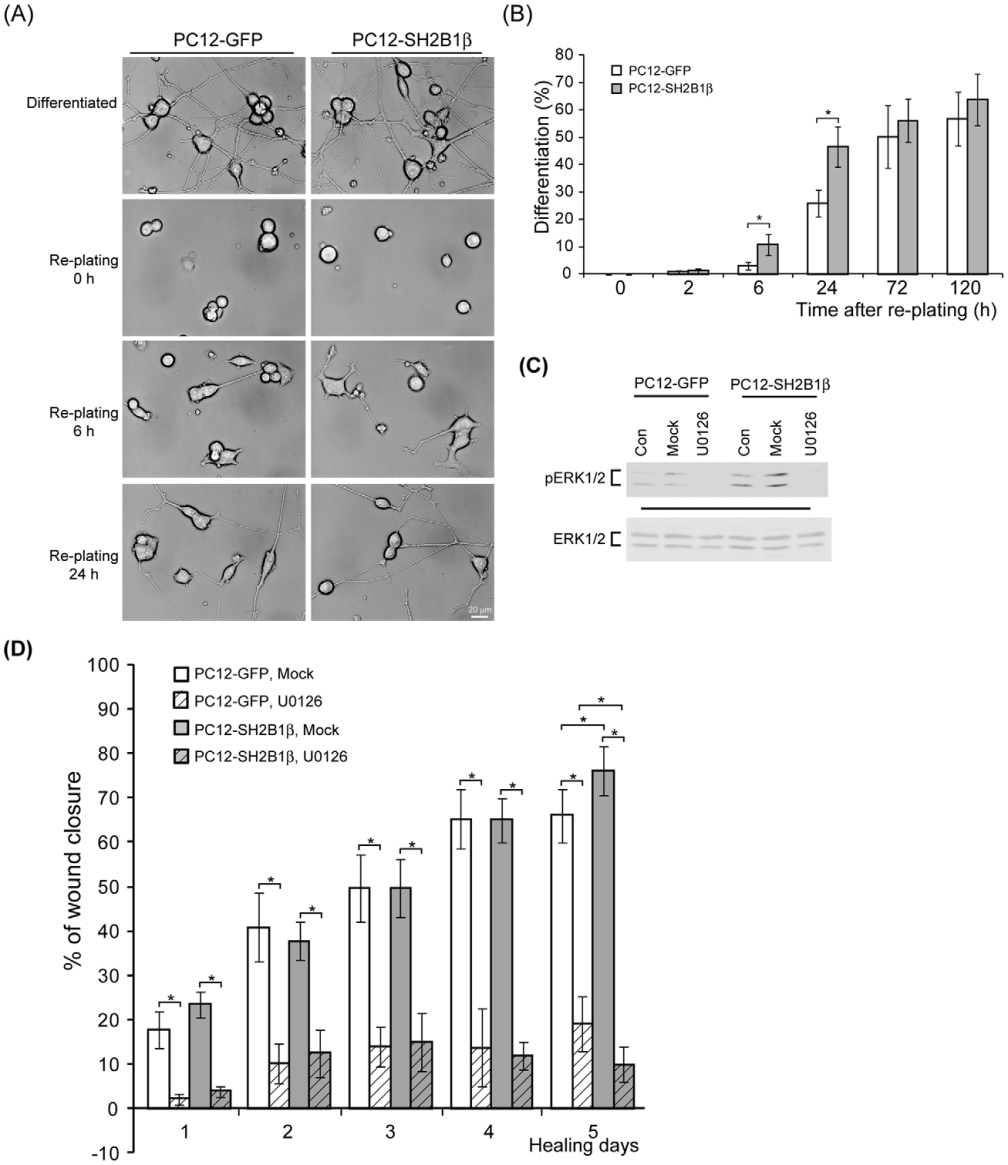
Overexpression of SH2B1β promotes pERK1/2-dependent neurite re-growth of differentiated PC12 cells after injury. (A) PC12-GFP and PC12-SH2B1β cells were differentiated as in Figure 1. On day 8, differentiated cells were trypsinized and neurites were removed via pippeting up and down. Live cell images of re-plated cells were taken over time. Representative images are shown. Scale bar: 20 μm. (B) Percentages of differentiated cells were determined as described in the Materials and Methods. Values are mean ± S.E.M. from four independent experiments. (*: P<0.05, paired Student's t-test) (C) PC12-GFP and PC12-SH2B1β cells were differentiated as in Figure 1. On day 8, cells were pre-treated with DMSO (Mock) or 20 μM U0126 for 1 h before wounding. Cell lysates were extracted on healing day 12 and resolved via SDS-PAGE followed by immunoblotting using antibodies against pERK1/2 and ERK1/2. (D) Percentages of wound closure were determined as in Figure 1. Values are mean ± S.E.M from at least three independent experiments. (*: P<0.05, paired Student's t-test).

### Cell culture and stable cell lines

The stock of PC12 cells was purchased from American Type Culture Collection. PC12 cells were maintained on the collagen-coated plates (coated with 0.16–0.2 mg/ml rat-tail collagen in 0.02 N acetic acid) and grown at 37°C in 10% CO_2_ in complete media, DMEM supplemented with 10% horse serum (HS), 5% fetal bovine serum (FBS), 1 mM L-glutamine and 1 mM antibiotic-antimycotic (Invitrogen). PC12 cells stably overexpressing GFP (PC12-GFP) or GFP-SH2B1β (PC12-SH2B1β) were made and cultured as described in Lin *et al* [42]. PC12 stable cell lines that express GFP-SH2B1β(Δ150–200) [PC12-(Δ150–200)] and GFP-SH2B1β(Δ200–268) [PC12-(Δ200–268)] were made for this study. A pooled population of stable clones was used to avoid clonal variation. During differentiation, cells were cultured in a differentiation medium (DMEM supplemented with 1% HS) containing NGF.

**Figure 3:**
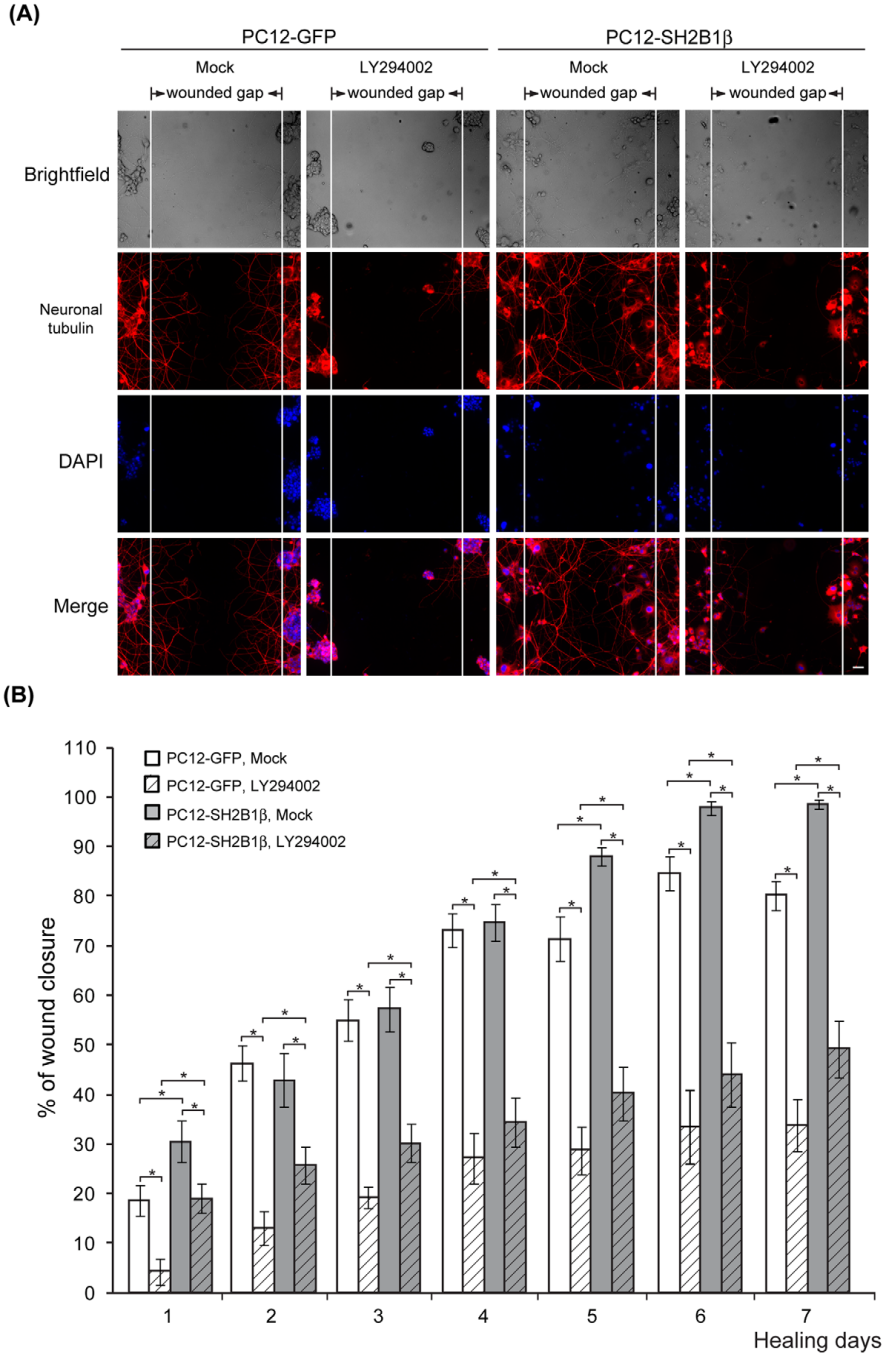
AKT-dependent neurite re-growth during wound healing of PC12 cells. PC12-GFP and PC12-SH2B1β cells were differentiated as in Figure 1. On day 8, differentiated cells were pretreated with DMSO (Mock) or 20 μM LY294002 for 1 h before wounding. (A) PC12-GFP and PC12-SH2B1β cells were fixed on healing day 7. Neuronal tubulin staining was determined using anti-Tuj1 antibody followed by Alexa Fluor 555 (red). DAPI (blue) staining showed the location of the nucleus. Scale bar: 50 μm. (B) Percentages of wound closure were determined as in Figure 1. Values are mean ± S.E.M. from four independent experiments. (*: P<0.05, paired Student's t-test).

### Neuronal injury experiments and cell migration tracking

For wound healing assays, cells were seeded on collagen-coated plates at 30% confluency and differentiated using NGF (50 ng/ml) for 8 days. The medium was changed every other day. The definition of neuronal differentiation for PC12 cells is that the length of neurites is at least twice the diameter of the cell body. On day 8, cells were wounded by scraping cells with a P10 pipette tip. Live cell images of cells were taken everyday using an inverted Zeiss Axiover 135 fluorescence microscope or the Carl Zeiss Observer Z1. The averaged width of the wounded gaps was measured from 12 different locations per wounded gap per time point (also see Figure S1). At least 6 wounded gaps were quantified from three independent experiments. The width of the remaining wounded gaps was averaged and divided by the width of initial wounded gaps. The percentage of wound closure was calculated as (100%- remaining wounded gaps%). The tracking function of AxioVision software (Zeiss) was used to track cell migration of a specific group of cells. For each wound healing experiment, at least 8 groups of cells per field were tracked for each time point. Position of cells was imaged and the net cell migration every 24 h was calculated for at least 6 days. Accumulated distance is the total distance that cells migrate over time (0–144 h). Cell motility is defined as the accumulated distance/time.

For re-plating experiments, PC12-GFP and PC12-SH2B1β cells were seeded on 10-cm collagen-coated plates at 30% confluency and differentiated with NGF (50 ng/ml). On day 8, cells were rinsed using 1× PBS three times, trypsinization, and centrifuged (235×g, 1 min at RT). Cells were re-suspended by pipetting up and down to remove the neurites, and then re-plated cells in 25 ng/ml NGF-containing differentiation medium. To examine neurite re-growth of differentiated PC12 cells, live cell images of random fields of at least 80 re-plated cells were taken using Carl Zeiss Observer Z1 microscope. The percentage of differentiated cells is defined as the numbers of cells with neurite length at least twice the diameter of cell bodies divided by the numbers of total cells counted.

### Immunoblotting and immunoprecipitation

Cells were harvested into RIPA (50 mM Tris, pH 7.5, 1% Triton X-100, 150 mM NaCl, 2 mM EGTA) containing 1 mM Na_3_VO_4_, 1 mM phenylmethanesulphonylfluoride (PMSF), 10 ng/ml aprotinin and 10 ng/ml leupeptin. Protein concentration of each sample was determined. Equal amounts of proteins were loaded to and resolved by sodium dodecyl sulfate-polyacrylamide gel electrophoresis (SDS-PAGE), followed by western blotting analysis using the indicated antibodies. For immunoprecipitation of PKC, cells were lysed in RIPA buffer containing 1 mM Na_3_VO_4_, 1 mM PMSF, 10 ng/ml aprotinin and 10 ng/ml leupeptin. Cell lysates were incubated with monoclonal PKC antibodies (1∶200) overnight at 4°C. Immunoprecipitated proteins were subjected to SDS-PAGE and Western blotting. The immunoblots were detected using either IRDye-conjugated IgG and the Odyssey Infrared Imaging System (LI-COR Biosciences) or the horseradish peroxidase-conjugated IgG and ECL system.

### Immunofluorescence staining

Cells were seeded on the collagen-coated plates at 30% confluency and differentiated with 50 ng/ml NGF in the differentiation medium for 8 days. On day 8, cells were wounded by a P10 pipette tip and fixed by 4% paraformaldehyde, permeabilized, and incubated with anti-neuronal tubulin antibodies, followed by incubation with the Alexa Fluor 555-conjugated secondary antibody for visualization. DAPI staining was used to visualize the nucleus. Images were taken by an upright fluorescent microscope Zeiss/Axioskop 2 mot plus and the Carl Zeiss Observer Z1.

### Inhibitor and activator assays

Cells were seeded on the collagen-coated 6-well plates at 30% confluency and differentiated with 50 ng/ml NGF in the differentiation medium for 8 days. On day 8, cells were pretreated with DMSO (Mock), 20 μM U0126, 20 μM LY294002 or 0.1–2.5 μM Bis for 1 h before wounding. Images were taken by the Carl Zeiss Observer Z1. For the PKC activation assay, wounded cells were treated with 162 nM PMA or DMSO (Mock). Images of cells were taken using the inverted Zeiss Axiover 135 fluorescence microscope or the Carl Zeiss Observer Z1. Images of wounded cells were tracked using the tracking function of Axio Vision software (Zeiss).

**Figure 4:**
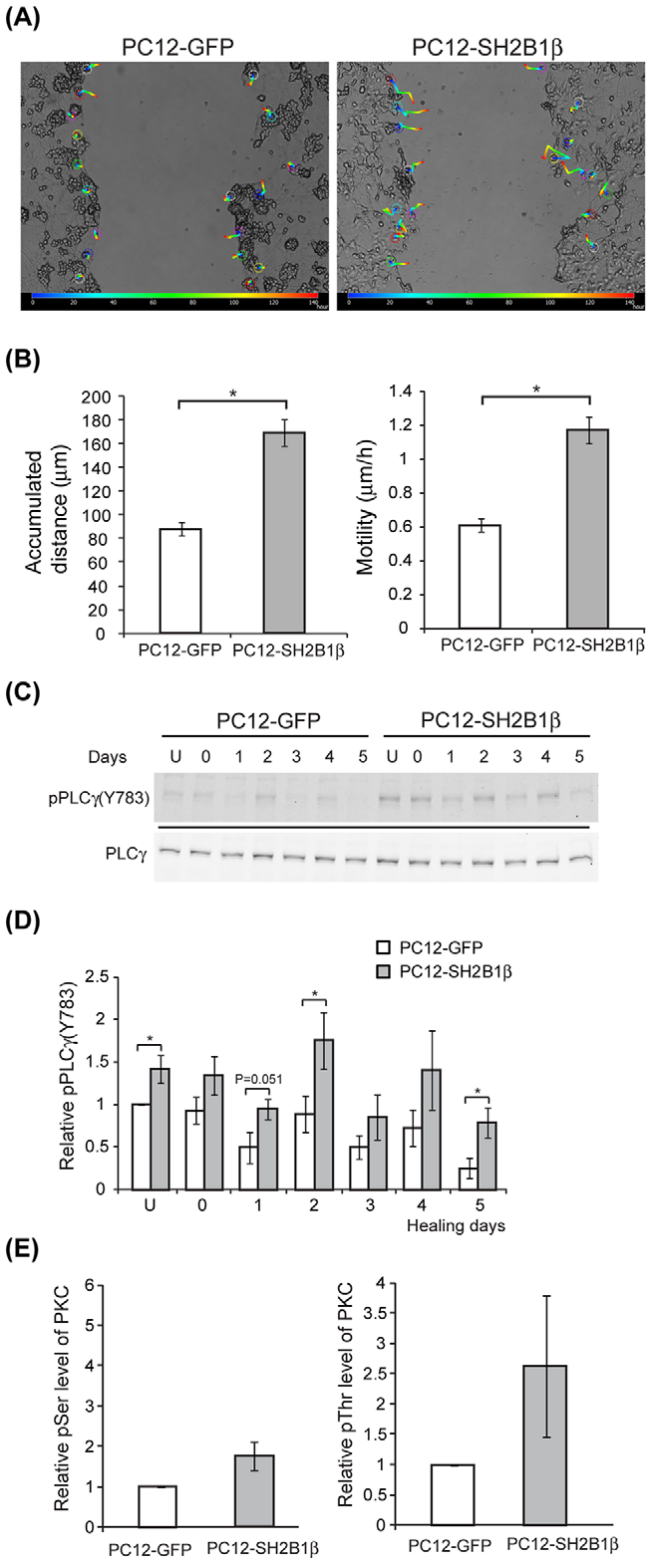
Increased cell migration of PC12-SH2B1β cells during wound healing associates with elevated pPLCγ1 and pPKC. PC12-GFP and PC12-SH2B1β cells were differentiated and subjected to wound healing as described in Figure 1. (A) Movement of cells around the wounded gaps was tracked as described in the Materials and Methods during healing for 6 days. The color lines represent the migration path of cells from time 0 (blue) to 144 h (red) during healing. (B) The accumulated distances and motility during time 0–144 h were calculated. Cell motility (rate of cell migration) is defined as accumulated distance/time. Values are mean ± S.E.M. from four independent experiments. (*: P<0.05, paired Student's t-test) (C) Equal amount of proteins from the harvested cell lysate of un-wounded (U) or wounded cells was resolved via SDS-PAGE and immunoblotted with anti-pPLCγ1 and anti-PLCγ1 antibodies. (D) Relative levels of pPLCγ1 were normalized to total PLCγ1 and then the levels in PC12-GFP cells on differentiated day 8 (U). Values are mean ± S.E.M. from at least three independent experiments. (*: P<0.05, one-way ANOVA) (E) Lysate or immunoprecipitated PKC were resolved via SDS-PAGE and immunoblotted with anti-PKC, pSer or pThr antibody. Relative pSer or pThr levels were normalized to immunoprecipitated PKC and then the levels in PC12-GFP cells on differentiated day 8 (un-wounded). Values are mean ± S.E.M. from three independent experiments for relative pSer levels and mean ± S.D. from two independent experiments for relative pThr levels.

**Figure 5:**
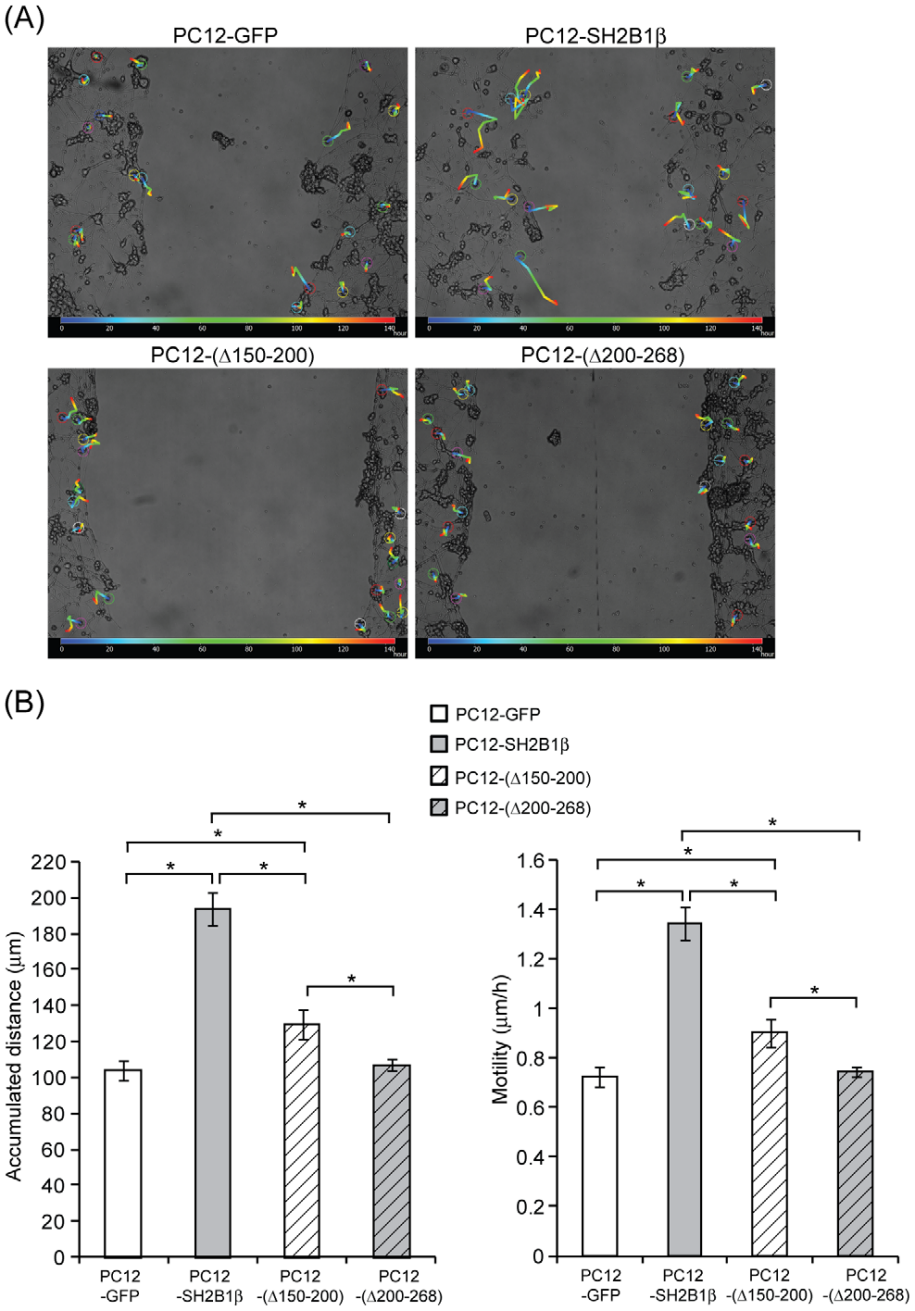
Overexpression of SH2B1β mutants reduce cell migration. PC12-GFP, PC12-SH2B1β, PC12-(Δ150–200) and PC12-(Δ200–268) cells were differentiated and subjected to wound healing as described in Figure 1. (A) Movement of cells around the wounded gaps was tracked as described in the Materials and Methods for 6 days during healing. The color lines represent the migration path of cells from time 0 (blue) to 144 h (red) during healing. (B) The accumulated distances and motility during time 0–144 h were calculated. Cell motility is defined as accumulated distance/time. Values are mean ± S.E.M. from three independent experiments. (*: P<0.05, paired Student's t-test).

**Figure 6:**
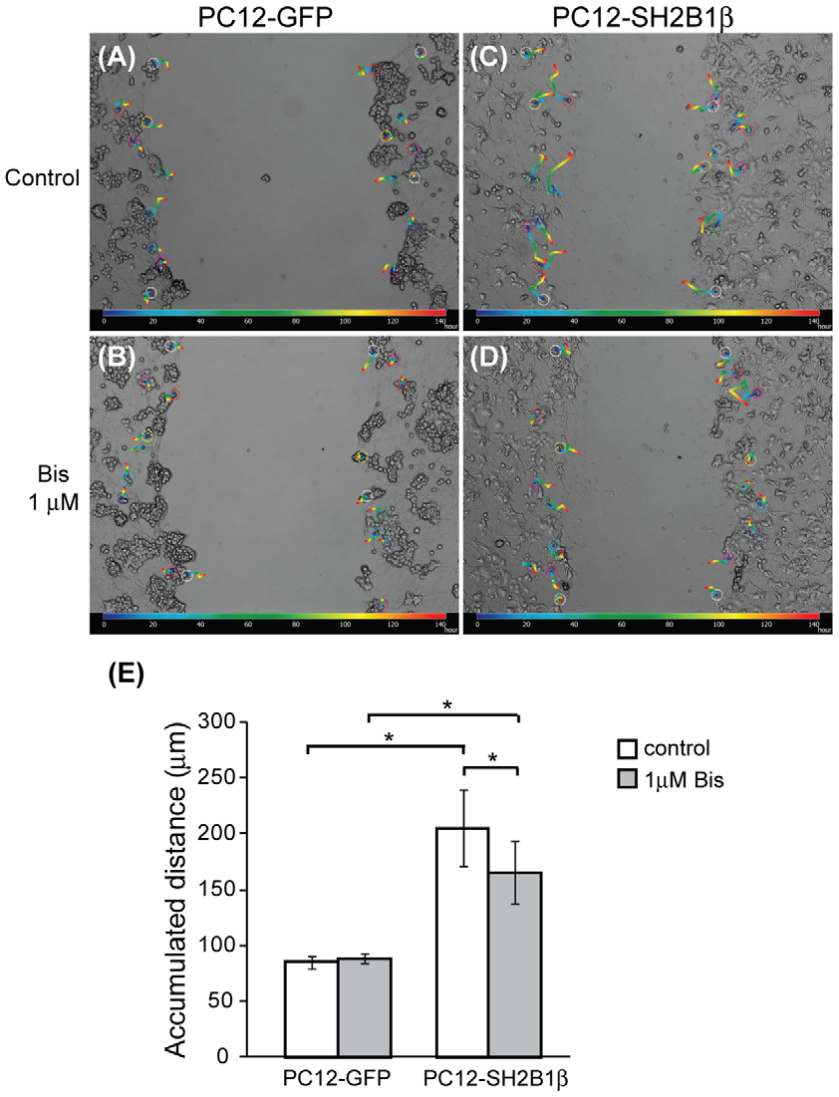
Inhibiting PKC activity decreases cell migration in PC12-SH2B1β cells. PC12-GFP and PC12-SH2B1β cells were differentiated as in Figure 1. On day 8, differentiated cells were pre-treated with or without 1 μM Bis for 1 h before wounding. (A–D) Migration paths were tracked from healing time 0 (blue) to 144 h (red) by tracking software. (E) Accumulated distance during healing days 0–6 for these cells were determined and shown. Values are mean ± S.E.M. from three independent experiments. (*: P<0.05, paired Student's t-test).

### Statistical analysis

Most of the comparisons were determined using paired Student's t-test or one-way ANOVA. Significant difference is defined as P value less than 0.05.

## Results

### Overexpression of SH2B1β promotes wound healing of differentiated PC12 cells

To determine the role of SH2B1β in neurite regeneration, two stable cell lines, PC12 cells stably expressing GFP (PC12-GFP) and PC12 cells stably expressing GFP-SH2B1β (PC12-SH2B1β), were established [42]. Wound healing (scraping) assays were performed to recapitulate injury and recovery. These two stable cell lines were differentiated into neurons by NGF (50 ng/ml) treatment for 8 days. On day 8, cells were subjected to mechanical scraping (Figure 1A and C). Differentiated PC12 cells depend on NGF for maintaining neuronal status. Thus, NGF was present at all time during healing (regeneration). The percentage of wound closure during healing was measured as described in the Materials and Methods, and in Figure S1. After healing for 5 days, differentiated PC12-SH2B1β cells healed better than PC12-GFP cells (Figure 1B and D). The quantified results revealed that the percentages of wound closure were higher for differentiated PC12-SH2B1β cells compared to those for PC12-GFP cells after healing day 4 (Figure 1E). On healing day 5, wound closure was 70% for differentiated PC12-GFP cells whereas it was 85% for differentiated PC12-SH2B1β cells. The percentage of wound closure for differentiated PC12-SH2B1β cells continued to increase over time while the healing rate was slower for PC12-GFP cells. To verify that the enhanced percentage of wound closure is SH2B1-dependent, knocking down SH2B1 seems to be the best approach to address the question. However, knocking down SH2B1 significantly reduces neuronal differentiation. Thus, two loss-of-function mutants of SH2B1β, SH2B1β(Δ150–200) and SH2B1β(Δ200–268), were used to examine the effect on neurite regeneration. SH2B1β(Δ150–200) lacks nuclear localizing sequences whereas SH2B1β(Δ200–268) lacks nuclear export signal. As shown in Figure 1F, PC12 cells stably expressing SH2B1β(Δ150–200) or SH2B1β(Δ200–268) reduced % of wound closure compared to cells expressing SH2B1β. These results suggest that the enhanced regeneration is SH2B1β-dependent. Notably, the wounded gaps were filled with neurites and a few cell bodies (Figure 1B and D). These results implicate the involvement of neurite re-growth and cell migration during the healing process. In addition, SH2B1β may promote neurite re-growth and/or cell migration during neuronal regeneration.

### Overexpression of SH2B1β promotes MEK-ERK1/2- and PI3K-AKT-dependent neurite re-growth

To examine whether SH2B1β could promote neurite re-growth, cell re-plating experiments were performed on the differentiated PC12 cells. PC12-GFP and PC12-SH2B1β cells were differentiated, trypsinized to detach cells, pipetted up and down to remove neurites and then re-plated. Neurite re-growth was monitored over time. As shown in Figure 2A, all neurites were removed after trypsinization. Neurite re-growth occurred as early as 2 h after re-plating. The percentages of differentiated cells (defined in the Materials and Methods) for PC12-SH2B1β cells at 6 and 24 h after re-plating were around 3.5- and 1.8-fold of those for PC12-GFP cells respectively (Figure 2B). Together with results from Figure 1, overexpression of SH2B1β enhances neurite re-growth in response to injury.

To determine what signaling molecules are involved in SH2B1β-mediated neurite re-growth, activity of MEK-ERK1/2 and PI3K-AKT pathways during neuronal regeneration were examined. PC12-GFP and PC12-SH2B1β cells were differentiated for 8 days and then pre-treated with U0126 (MEK inhibitor) or LY294002 (PI3K inhibitor) for 1 h before wounding. In the present of U0126, the phosphorylation of ERK1/2 was inhibited (Figure 2C). The percentages of wound closure were reduced 70–85% for PC21-GFP cells and 58–86% for PC12-SH2B1β cells in the presence of U0126 (Figure 2D). These results suggest that ERK1/2 activity is essential for neurite re-growth as well as SH2B1β-mediated functions.

**Figure 7:**
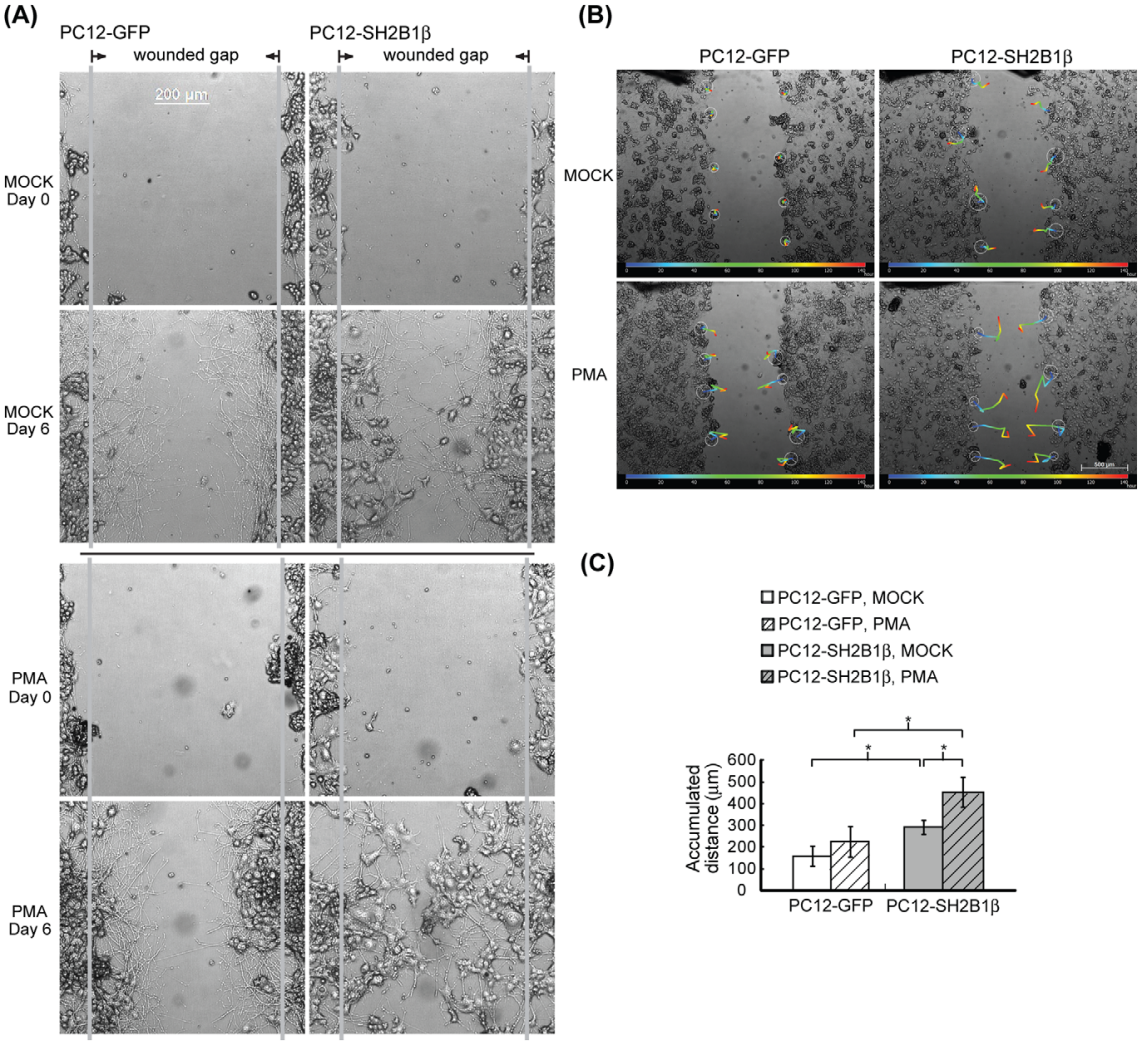
PMA increases SH2B1β-enhanced cell migration during wound healing. PC12-GFP and PC12-SH2B1β cells were differentiated as in Figure 1. On day 8, cells were wounded, treated without or with 162 nM PMA and allowed to heal for 6 days. (A) Live cell images were taken using the inverted Zeiss Axiover 135 fluorescence microscope or the Carl Zeiss Observer Z1. Representative images are shown from three independent experiments. (B) Migration paths were tracked from healing time 0 (blue) to 144 h (red) by tracking software every 24 h. (C) Accumulated distance during healing days 0–6 were determined and shown. Values are mean ± S.E.M. from three independent experiments. (*: P<0.05, paired Student's t-test).

PI3K-AKT pathway contributes to both cell survival and neurite outgrowth [11], [23], [50]. The effects of inhibiting phosphorylated AKT during wound healing were also examined. Tuj1 staining results showed that neurite re-growth was better for differentiated PC12-SH2B1β cells compared to PC12-GFP cells on healing day 7. In addition, LY294002 treatment reduced neurite re-growth (Figure 3A). Similar with the inhibition of pERK1/2, neurite re-growth was reduced 37 to 77% in the present of LY294002 for both cell lines (Figure 3B). These results demonstrated that both MEK-ERK1/2 and PI3K-AKT pathways are required for SH2B1β-mediated enhancement of neurite re-growth.

**Figure 8:**
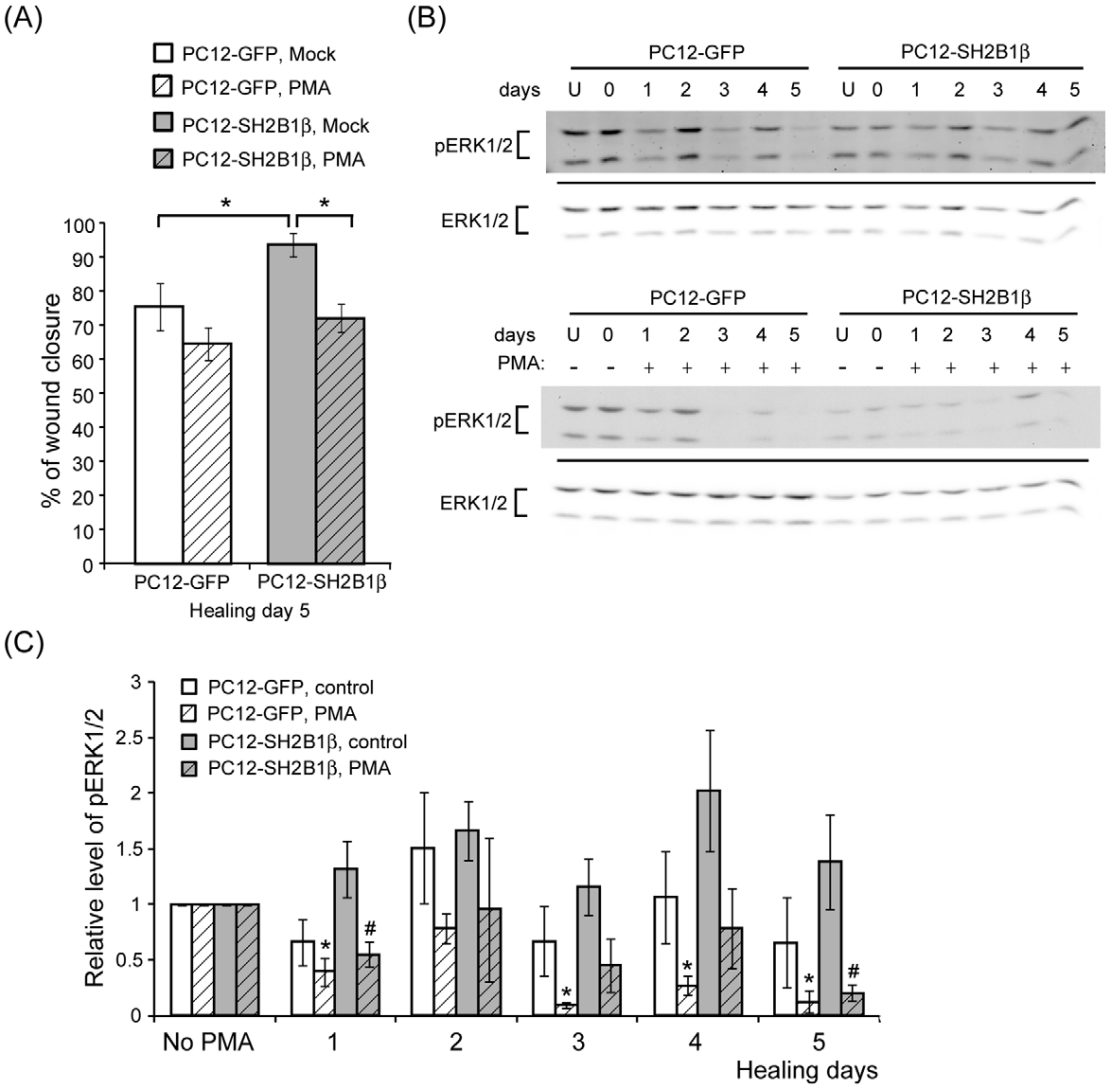
PMA treatment reduces wound closure and pERK1/2. PC12-GFP and PC12-SH2B1β cells were differentiated as in Figure 1. (A) On day 8, differentiated cells were wounded and treated with DMSO (Mock) or 162 nM PMA. Percentages of wound closure on healing day 5 were determined as described in the Materials and Methods. Values are mean ± S.E.M. from three independent experiments. (*: P<0.05, Student's t-test) (B) On day 8, un-wounded (U) or wounded cells were treated with or without 162 nM PMA. Cell lysates were collected from un-wounded (U) cells and cells during healing days 0–5. Equal amount of proteins from lysates was resolved via SDS-PAGE and immunoblotted with anti-pERK1/2 and anti-ERK1/2 antibodies. (C) Relative pERK1/2 levels with or without PMA treatment were normalized to total ERK1/2 and then the levels of those on differentiated day 8. Values are mean ± S.E.M. from at least three independent experiments. (*: compared to the levels in no PMA-treated PC12-GFP cells on differentiated day 8; #: compared to the levels in no PMA-treated PC12-SH2B1β cells on differentiated day 8, P<0.05, paired Student's t-test).

**Figure 9:**
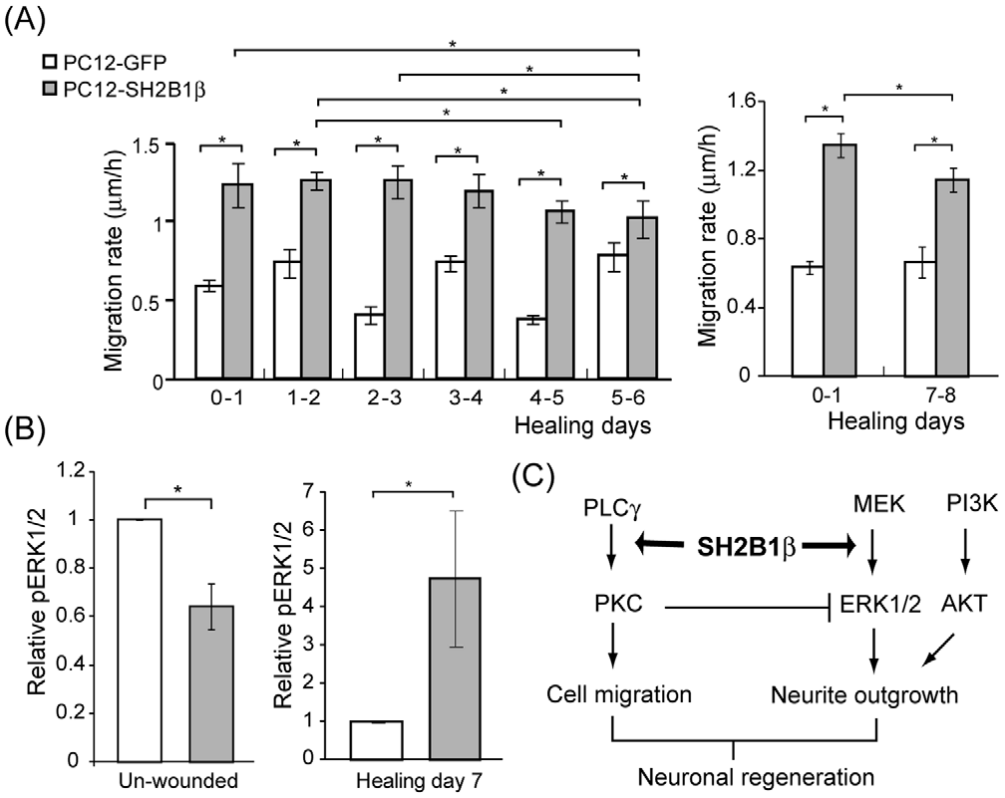
Motility of cells overexpressing SH2B1β is higher during healing days 0–4 and reduced during healing days 5–8. PC12-GFP and PC12-SH2B1β cells were differentiated and subjected to wound healing as described in Figure 1. (A) Cell migration was tracked every day and the net distance per day was calculated. Values are mean ± S.E.M. from four independent experiments for healing days 0–6 (left panel) and three independent experiments for healing days 0–1 and 7–8 (right panel). (*: P<0.05, paired Student's t-test) (B) Relative pERK1/2 levels were normalized to total ERK1/2 and then the levels in PC12-GFP cells on differentiated day 8 (Un-wounded) or on healing day 7. Values are mean ± S.E.M. from eighteen independent experiments for un-wounded cells and four independent experiments for healing day 7. (*: P<0.05, paired Student's t-test) (C) SH2B1β modulates PKC-dependent cell migration and ERK1/2-mediated neurite re-growth depending on the healing stages. PKC-dependent cell migration antagonizes ERK1/2-mediated neurite re-growth, and fine temporal regulation of cell migration and neurite re-growth contributes to the optimal healing outcome.

### Overexpression of SH2B1β correlates with enhanced cell migration and elevated phosphorylation of PLCγ1 and PKC

In addition to regenerated neurites, cell bodies also appeared in the wounded gaps for both PC12-GFP and PC12-SH2B1β cells on healing day 5, indicative of cell migration to the wounded gaps (Figure 1B and D). We thus examined whether overexpression of SH2B1β would enhance cell migration to promote regeneration. PC12-GFP and PC12-SH2B1β cells were differentiated and subjected to wound healing protocol. Migration of cells from healing time 0 (blue color) to 144 h (red color) were tracked and analyzed (Figure 4A). During 6 days of healing, the accumulated distance that PC12-SH2B1β cells migrated was 170 µm in average, compared to 90 µm by PC12-GFP cells (Figure 4B, left panel), suggesting that their motility was higher (Figure 4B, right panel). Based on the known effects of PKC on cell motility, we next examined whether SH2B1β increases cell migration through PLCγ and/or PKC. To this end, cell lysates were collected from differentiated PC12-GFP and PC12-SH2B1β cells before wounding (U) and during healing days 0–5. Phosphorylation of PLCγ1 was determined via western blotting. The overall phosphorylation levels of PLCγ1 in differentiated PC12-SH2B1β cells were around 1.4- to 3.1-fold of those in PC12-GFP cells. In addition, the levels of pPLCγ1 were reduced on healing day 5 (Figure 4C–D). The phosphorylation of PKC, a downstream effector of PLCγ1, was also examined. Total PKC was immunoprecipitated and its phosphorylation level was determined. The relative PKC phosphorylations at serine and threonine residues for differentiated PC12-SH2B1β cells were around 1.7- and 2.6-fold of those in PC12-GFP cells respectively (Figure 4E). These results are consistent with our hypothesis that SH2B1β promotes cell migration through PLCγ-PKC pathway. To determine whether the enhanced cell migration is SH2B1-dependent, cell migration after scraping injury was determined using PC12-(Δ150–200) and PC12-(Δ200–268) cells that express two loss-of-function mutants of SH2B1β. Cell migration for PC12-(Δ150–200) and PC12-(Δ200–268) cells during healing was reduced compared to PC12-SH2B1β cells and comparable to control PC12-GFP cells (Figure 5). Along this line, cell migration for PC12-SH2B1β cells was reduced around 20% by 1 μM PKC inhibitor, Bisindolyleimide I (Bis) (Figure 6). Dose-dependent inhibition of cell migration by 0–2.5 μM Bis was determined (Figure S2). To reduce the possibility that inhibiting PKC may affect normal physiology of neuron cells, low dosage of 1 μM Bis was used for this study. The fact that cell migration of PC12-GFP cells was not inhibited by 1 μM Bis could be due to lower motility or PKC activity in PC12-GFP cells (Figure 4). Together, these results implicate that PLCγ-PKC pathway is involved in SH2B1β-mediated cell migration.

### PKC activation promotes SH2B1β-enhanced cell migration

Since PKC activity is involved in SH2B1β-enhanced cell migration during wound healing, we next determined whether activation of PKC contributes to SH2B1β-mediated migration. Differentiated PC12-GFP and PC12-SH2B1β cells were wounded, mock-treated or treated with PKC activator – phorbol 12-myristate 13-acetate (PMA), and the effect on cell migration was assessed. PMA activates PKC because of its similar structure to DAG. The distance PC12-SH2B1β cells migrated was around 2-fold of that by PC12-GFP cells in mock-treated conditions (Figure 7). PMA treatment significantly enhanced cell migration of PC12-SH2B1β cells after healing for 6 days (Figure 7). The fact that PMA stimulation promoted obvious cell migration for PC12-SH2B1β cells but not so much for PC12-GFP cells suggests that overexpression of SH2B1β may recruit PMA substrates closer to plasma membrane where PMA acts. To confirm that the activation of PKC was induced after the treatment of PMA, the differentiated PC12-GFP and PC12-SH2B1β cells were treated with or without PMA. Relative phosphoserine (pSer) of PKC was increased approximately 60% after 6 h of PMA treatment for PC12-GFP but not for PC12-SH2B1β cells (Figure S3). Relative phosphothreonine (pThr) of PKC, on the other hand, was induced 1.1- to 3.4-fold for both cell lines (Figure S3). PMA-induced PKC phosphorylation peaked at 6 h and then reduced by 24 h. Consistent with the results in Figure 4E, basal phosphorylation of PKC was higher in PC12-SH2B1β cells compared to PC12-GFP cells. Interestingly, PMA treatment also increased the expression of PKC. The increased PKC levels in differentiated PC12-SH2B1β cells were 1.3- to 1.6-fold of those in PC12-GFP cells (Figure S3). This may explain why the relative phosphorylation of PKC (after normalized to total PKC levels) in response to PMA stimulation was not higher for PC12-SH2B1β cells (Figure S3). A study showed that PMA promoted degradation of PKC in human osteosarcoma cells [51]. However, it was not clear which isozyme was referred to. Our data suggested that PMA promoted degradation of PKCδ (data not shown) but increased synthesis of pan-PKCs (Figure S3). Thus, PMA-increased expression and phosphorylation of PKC likely account for the increased cell migration of PC12-SH2B1β cells.

### Fine balance between cell migration and neurite re-growth warrants better wound healing

As PMA treatment promotes cell migration during wound healing, it unexpectedly interfered with wound closure. Treatment of PMA reduced 10% wound closure for differentiated PC12-GFP cells and 25% for differentiated PC12-SH2B1β cells (Figure 8A). This result raises a possibility that high motility of cells may impede neurite outgrowth. Because neurite re-growth is largely dependent on pERK1/2 (Figure 2), we thus examine whether pERK1/2 levels would be affected by PMA treatment. Cell lysates were collected from differentiated cells before wounding (U) and during healing days 0–5. Phosphorylation of ERK1/2 was examined via western blotting. As shown in Figure 8B–C, pERK1/2 levels were significantly reduced after the treatment of PMA for both PC12-GFP and PC12-SH2B1β cells. The down-regulation of pERK1/2 levels likely contributes to the reduced neurite re-growth and wound healing. This result also reveals an antagonistic role of cell motility and neurite outgrowth. As cell migration is enhanced by PMA treatment, neurite re-growth is reduced. If this is true, higher cell motility of PC12-SH2B1β cells may not accompany with better neurite re-growth. Taking a closer look at cell migration each day for both cell lines, cell motility for PC12-SH2B1β cells was higher during healing days 0–4 and reduced after healing day 4 (Figure 9A, left panel). Cell migration during healing day 7–8 was also reduced compared to healing day 0–1 for PC12-SH2B1β cells (Figure 9A, right panel). On the other hand, pERK1/2 levels were lower for un-wounded PC12-SH2B1β cells compared to PC12-GFP cells (Figure 9B, left panel), whereas they were higher on healing day 7 (Figure 9B, right panel). The reverse correlation between cell migration and pERK1/2 suggests that overexpression of SH2B1β significantly promoted cell migration toward wounded area before healing day 4 and the neurite outgrowth after healing day 4. These results suggest that a fine balance between cell motility and neurite re-growth warrants better healing.

Together, results from this study demonstrated that overexpression of SH2B1β promoted regeneration of differentiated PC12 cells, in response to scraping injury, after healing day 4. Rate of SH2B1β-enhanced cell migration was faster during healing days 0–4 and pERK1/2-mediated neurite re-growth was increased after healing day 4. The fact that activated PKC increased cell migration but reduced neurite re-growth suggests that cell migration and neurite re-growth do not occur concomitantly during regeneration.

## Discussion

In this study, we demonstrated that overexpression of a signaling adaptor protein SH2B1β promotes neurite regeneration of differentiated PC12 cells. During neurite regeneration, overexpression of SH2B1β increases both cell motility and neurite re-growth (Figures 1, 2 and 4). Neurite re-growth is significantly blocked in the presence of MEK or PI3K inhibitor for both PC12-GFP and PC12-SH2B1β cells (Figures 2 and 3). No obvious effect on cell migration was found in the presence of these two inhibitors. These results are in line with published results that both ERK1/2 and AKT contribute to neurite outgrowth [10], [25].

Accumulating evidence from behavior, genetic and pharmacological studies have implicated PKC in an array of neuronal functions, including release of neurotransmitters, synaptic functions, learning and memory processes [52], [53], [54]. However, the effect of PKC activation on neurite outgrowth has been controversial. For example, PKC inhibitors have been reported to block neurite outgrowth in retinal axons, dorsal root ganglion neurons, sympathetic neurons, PC12 cells, and hippocampal cultures [55], [56], [57], [58], [59]. PKC inhibitors have also been shown to promote dendritic growth of Purkinjie cells in cerebellar slice cultures and to extend filopodia in DRG [60], [61]. The addition of dopamine causes neurite retraction of retinal neurons via activating PKC, and eicosanoid activation of PKC also serves as growth cone repellent signaling [62], [63]. The discrepancy of these results could simply be due to various types of neurons from peripheral or central nervous systems. Alternatively, as suggested in this study, temporal and possibly spatial control of PKC activity during wound healing lead to diverse cell fates or healing outcomes. Through inhibitor and activator assays, we further demonstrated that PLCγ-PKC pathway was involved in SH2B1β-enhanced cell migration (Figures 6–7). Although PMA is a well-known activator of PKC, Src has been suggested to be one of the downstream targets and has been implicated in cell migration [64], [65], [66]. To address this possibility, Src phosphorylation (pSrc) with or without PMA treatment was investigated. As shown in Figure S4, with PMA treatment, levels of pSrc were significantly increased around 55 and 67% for PC12-GFP cells on healing days 1 and 4, respectively. However, cell migration of PC12-GFP cells was not significantly affected by PMA (Figure 7C). For PC12-SH2B1β cells, pSrc was significantly increased by PMA only on healing day 5 (Figure S4). However, PMA treatment increased migration of PC12-SH2B1β cells before healing day 5 (Figure 7). Thus, it seems unlikely that PMA stimulates SH2B1β-mediated cell migration through increasing pSrc.

We found that cell motility was reversely correlated with pERK1/2-dependent neurite re-growth. After healing day 4, cell motility was reduced for PC12-SH2B1β cells whereas the wound closure was better (Figures 1 and 9). Nonetheless, cell motility is not necessary further reduced afterwards because, at a later healing stage – healing day 23, more cell bodies were found at wounded gaps suggesting another phase of increased cell migration (Figure S5). Thus, we think that as both cell migration and neurite re-growth contribute to neuronal regeneration, these processes may occur in a pulsatile manner. An intriguing question raised here is what signals cell to reduce or increase cell migration. We found that PMA treatment increased the levels of SH2B1β (Figure S6), phosphorylation of PKC (Figure S3), and cell migration (Figure 7). Although SH2B1β has predicted PKC phosphorylation site, exactly how PKC activity and SH2B1β regulate each other during neuronal regeneration and whether putative regulators are involved remain to be elucidated.

Evidence suggests that injury can induce stem cells migrating to the injured sites to aid wound healing [67], [68], [69], [70]. Thus, promoting migration of neural stem cells or neural progenitor cells to injured sites can enhance regeneration. As we demonstrated that overexpression of SH2B1β promoted cell migration, we cannot discern whether SH2B1β promotes differentiation of PC12 cells and then their migration to the wounded area, or SH2B1β promotes cell migration to the wounded area and then enhances their differentiation. Unfortunately, it is difficult to track undifferentiated cells from a distance to the wounded area in our system.

The current study reveals a novel function of SH2B1β in promoting neuronal regeneration. MEK-ERK1/2 and PI3K-AKT pathways are necessary for SH2B1β-mediated neurite re-growth during wound healing. Increased SH2B1β level associates with elevated phosphorylation of PLCγ and PKC, and thus promotes cell migration during regeneration. We further demonstrate that a cross-talk between PKC and ERK1/2 pathways contributes to temporal regulation of cell migration, neurite re-growth and thus the outcome of regeneration.

## Supporting Information

Figure S1 **The quantification of wound closure.** Distances of at least 12 different locations within each wounded gap were measured. The percentages of remaining wounded gaps were calculated by averaged width of the wounded gap per time point divided by the wounded gap on healing day 0. The percentage of wound closure was defined as 100%- remaining wounded gaps%.
(DOC)

Figure S2 **Dose-dependent inhibition of cell migration by PKC inhibition.** PC12-GFP and PC12-SH2B1β cells were differentiated as in Figure 1. On day 8, differentiated cells were pre-treated with or without 1 μM or 2.5 μM Bis for 1 h before wounding. Accumulated distance during healing days 0–6 and net distance between healing days 0 and 6 of cell migration were determined and shown. Values are mean ± S.E.M. from four independent experiments for control cells and mean ± S.D. from two independent experiments for Bis-treated cells. (*: P<0.05 paired Student's t-test).
(DOC)

Figure S3 **Overexpression of SH2B1β enhances PMA-induced PKC phosphorylation and synthesis.** PC12-GFP and PC12-SH2B1β cells were differentiated. On day 8, differentiated cells were treated with or without 162 nM PMA and harvested on indicating time points. Total lysates or immunoprecipitated PKCs were resolved via SDS-PAGE and immunoblotted with anti-PKC, pSer, pThr or ERK1/2 antibody. Relative pSer or pThr levels were normalized to the amount of immunoprecipitated PKC and then the levels in PC12-GFP cells on differentiated day 8 (time 0 h). Relative PKC levels of cell lysates were normalized to ERK1/2 and then the level in PC12-GFP cells on differentiated day 8 (time 0 h).
(DOC)

Figure S4 **Src may participate in PMA-mediated cell migration of PC12 cells.** PC12-GFP and PC12-SH2B1β cells were differentiated and subjected to wound healing as described in Figure 1. (A) Equal amount of proteins from the lysates of un-wounded (U) or cells during healing days 0–5 was resolved via SDS-PAGE and immunoblotted with anti-pSrc(Y416) and anti-Src antibodies. (B) Relative levels of pSrc were normalized to total Src and then the levels in PC12-GFP cells on differentiated day 8 (U). Values are mean ± S.E.M. from at least three independent experiments. (*: P<0.05, one-way ANOVA) (C) Relative levels of pSrc were normalized to total Src and then the levels in PC12-GFP cells on healing day 7. Values are mean ± S.E.M. from five independent experiments. (*: P<0.05, paired Student's t test) Anti-pSrc(Tyr416) antibody reacts with Src family kinases [71].
(DOC)

Figure S5 **Overexpression of SH2B1β increases cell migration.** PC12-GFP and PC12-SH2B1β cells were differentiated and subjected to wound healing as described in Figure 1. Live cell images were taken on healing days 0 and 23 using Carl Zeiss Observer Z1 microscope. Scale bar: 200 μm.
(DOC)

Figure S6 **PMA treatment increases SH2B1β levels.** PC12-SH2B1β cells were differentiated and subjected to wound healing protocol. On differentiation day 8, un-wounded (U) or wounded cells were treated with or without 162 nM PMA. Cell lysates were collected from un-wounded (U) cells and cells during healing days 0–5. Equal amount of proteins from lysates was resolved via SDS-PAGE and immunoblotted with anti-SH2B1 and anti-HDAC antibodies. Polyclonal antibody to rat SH2B1 was generously provided by Dr. Christin Carter-Su at the University of Michigan, USA.
(DOC)
