# Supplementary material for: Interplay between Cell Migration and Neurite Outgrowth Determines SH2B1β-Enhanced Neurite Regeneration of Differentiated PC12 Cells
Source: PLoS One. 2012 Apr 23;7(4):e34999. doi: 10.1371/journal.pone.0034999 (PMC3335126; doi:10.1371/journal.pone.0034999)
Supplement: Figure S1 — The quantification of wound closure. Distances of at least 12 different locations within each wounded gap were measured. The percentages of remaining wounded gaps were calculated by averaged width of the wounded gap per time point divided by the wounded gap on healing day 0. The percentage of wound closure was defined as 100%- remaining wounded gaps%. (DOC) [file pone.0034999.s001.doc]

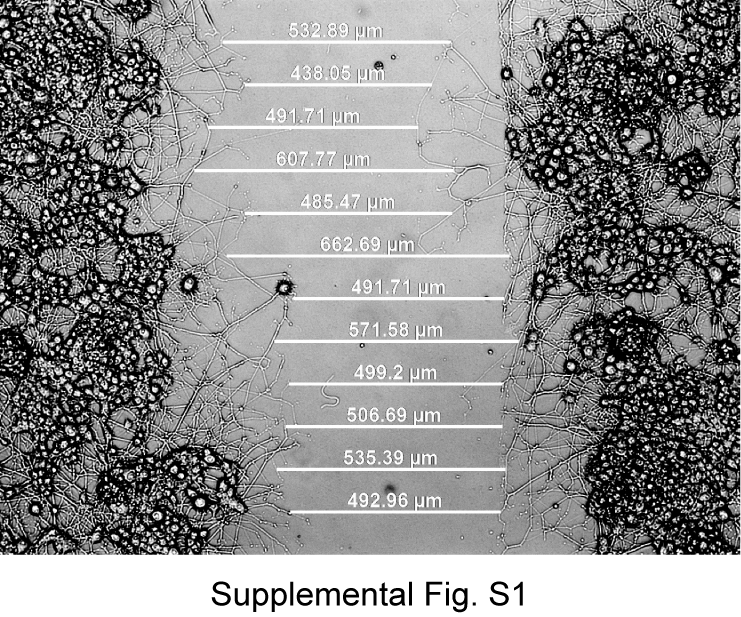


**Figure S1 The quantification of wound closure**

Distances of at least 12 different locations within each wounded gap were measured. The percentages of remaining wounded gaps were calculated by averaged width of the wounded gap per time point divided by the wounded gap on healing day 0. The percentage of wound closure was defined as 100%- remaining wounded gaps%.
