# Supplementary material for: Interplay between Cell Migration and Neurite Outgrowth Determines SH2B1β-Enhanced Neurite Regeneration of Differentiated PC12 Cells
Source: PLoS One. 2012 Apr 23;7(4):e34999. doi: 10.1371/journal.pone.0034999 (PMC3335126; doi:10.1371/journal.pone.0034999)
Supplement: Figure S2 — Dose-dependent inhibition of cell migration by PKC inhibition. PC12-GFP and PC12-SH2B1β cells were differentiated as in Figure 1. On day 8, differentiated cells were pre-treated with or without 1 μM or 2.5 μM Bis for 1 h before wounding. Accumulated distance during healing days 0–6 and net distance between healing days 0 and 6 of cell migration were determined and shown. Values are mean ± S.E.M. from four independent experiments for control cells and mean ± S.D. from two independent experiments for Bis-treated cells. (*: P<0.05 paired Student's t-test). (DOC) [file pone.0034999.s002.doc]

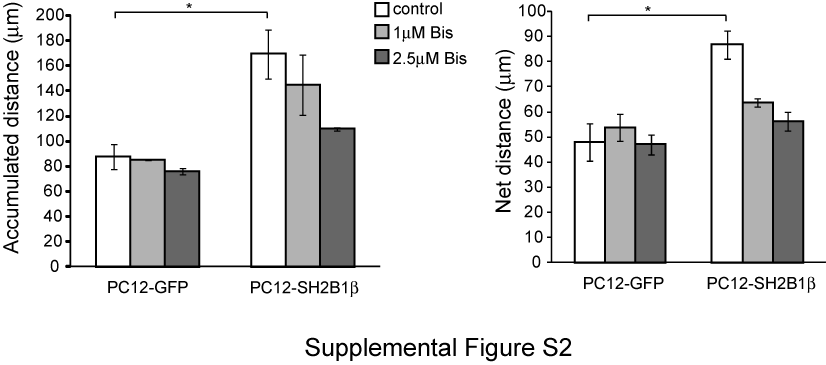


**Figure S2 Dose-dependent inhibition of cell migration by PKC inhibition**

PC12-GFP and PC12-SH2B1β cells were differentiated as in Figure 1. On day 8,

differentiated cells were pre-treated with or without 1 μM or 2.5 μM Bis for 1 h

before wounding. Accumulated distance during healing days 0-6 and net distance

between healing days 0 and 6 of cell migration were determined and shown. Values

are mean ± S.E.M. from four independent experiments for control cells and mean ± S.D. from two independent experiments for Bis-treated cells. (*: P < 0.05 paired Student’s t-test)
