# Supplementary material for: Interplay between Cell Migration and Neurite Outgrowth Determines SH2B1β-Enhanced Neurite Regeneration of Differentiated PC12 Cells
Source: PLoS One. 2012 Apr 23;7(4):e34999. doi: 10.1371/journal.pone.0034999 (PMC3335126; doi:10.1371/journal.pone.0034999)
Supplement: Figure S3 — Overexpression of SH2B1β enhances PMA-induced PKC phosphorylation and synthesis. PC12-GFP and PC12-SH2B1β cells were differentiated. On day 8, differentiated cells were treated with or without 162 nM PMA and harvested on indicating time points. Total lysates or immunoprecipitated PKCs were resolved via SDS-PAGE and immunoblotted with anti-PKC, pSer, pThr or ERK1/2 antibody. Relative pSer or pThr levels were normalized to the amount of immunoprecipitated PKC and then the levels in PC12-GFP cells on differentiated day 8 (time 0 h). Relative PKC levels of cell lysates were normalized to ERK1/2 and then the level in PC12-GFP cells on differentiated day 8 (time 0 h). (DOC) [file pone.0034999.s003.doc]

**
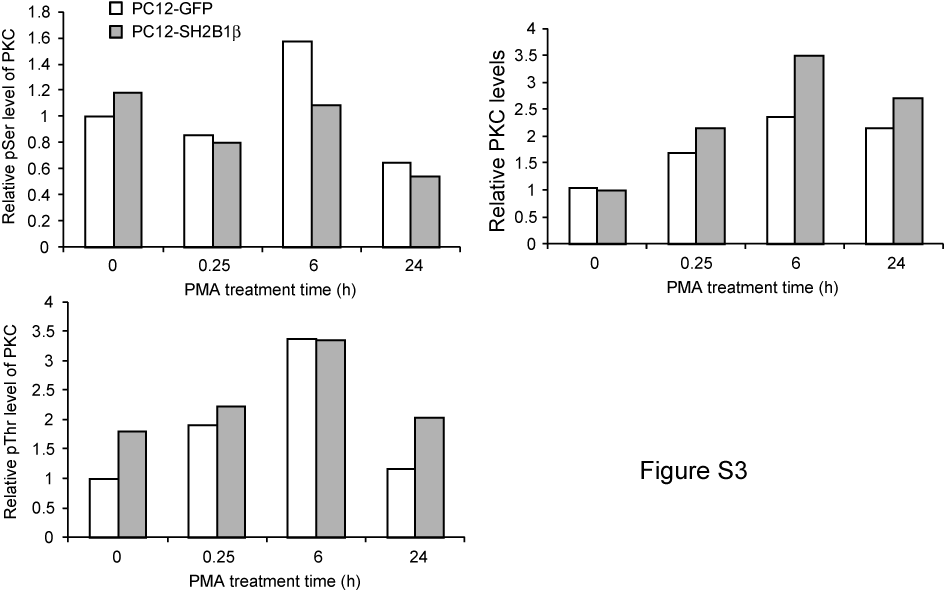
**

**Figure S3 Overexpression of SH2B1β enhances PMA-induced PKC phosphorylation and synthesis**

PC12-GFP and PC12-SH2B1β cells were differentiated. On day 8, differentiated cells were treated with or without 162 nM PMA and harvested on indicating time points. Total lysates or immunoprecipitated PKCs were resolved via SDS-PAGE and immunoblotted with anti-PKC, pSer, pThr or ERK1/2 antibody. Relative pSer or pThr levels were normalized to the amount of immunoprecipitated PKC and then the levels in PC12-GFP cells on differentiated day 8 (time 0 h). Relative PKC levels of cell lysates were normalized to ERK1/2 and then the level in PC12-GFP cells on differentiated day 8 (time 0 h).
