# Supplementary material for: Interplay between Cell Migration and Neurite Outgrowth Determines SH2B1β-Enhanced Neurite Regeneration of Differentiated PC12 Cells
Source: PLoS One. 2012 Apr 23;7(4):e34999. doi: 10.1371/journal.pone.0034999 (PMC3335126; doi:10.1371/journal.pone.0034999)
Supplement: Figure S4 — Src may participate in PMA-mediated cell migration of PC12 cells. PC12-GFP and PC12-SH2B1β cells were differentiated and subjected to wound healing as described in Figure 1. (A) Equal amount of proteins from the lysates of un-wounded (U) or cells during healing days 0–5 was resolved via SDS-PAGE and immunoblotted with anti-pSrc(Y416) and anti-Src antibodies. (B) Relative levels of pSrc were normalized to total Src and then the levels in PC12-GFP cells on differentiated day 8 (U). Values are mean ± S.E.M. from at least three independent experiments. (*: P<0.05, one-way ANOVA) (C) Relative levels of pSrc were normalized to total Src and then the levels in PC12-GFP cells on healing day 7. Values are mean ± S.E.M. from five independent experiments. (*: P<0.05, paired Student's t test) Anti-pSrc(Tyr416) antibody reacts with Src family kinases [71]. (DOC) [file pone.0034999.s004.doc]

**
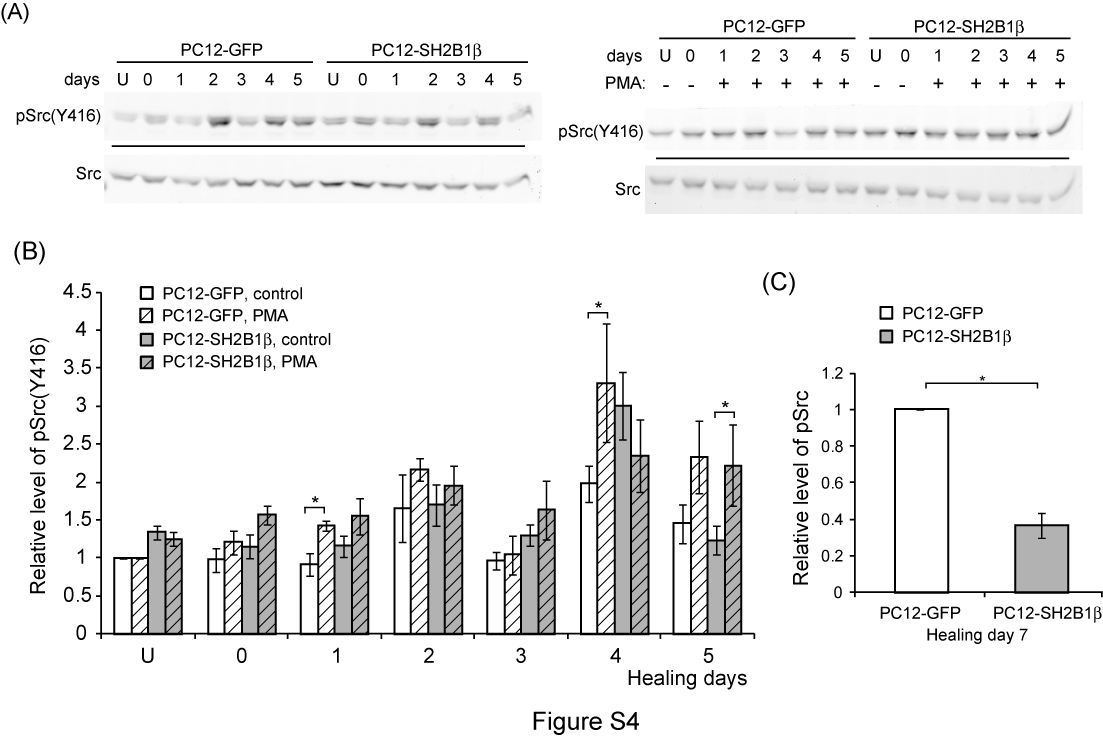
**

**Figure S4 Src may participate in PMA-mediated cell migration of PC12 cells**

PC12-GFP and PC12-SH2B1β cells were differentiated and subjected to wound healing as described in Figure 1. (A) Equal amount of proteins from the lysates of un-wounded (U) or cells during healing days 0-5 was resolved via SDS-PAGE and immunoblotted with anti-pSrc(Y416) and anti-Src antibodies. (B) Relative levels of pSrc were normalized to total Src and then the levels in PC12-GFP cells on differentiated day 8 (U). Values are mean ± S.E.M. from at least three independent experiments. (*: P < 0.05, one-way ANOVA) (C) Relative levels of pSrc were normalized to total Src and then the levels in PC12-GFP cells on healing day 7. Values are mean ± S.E.M. from five independent experiments. (*: P < 0.05, paired Student’s t test)

Anti-pSrc(Tyr416) antibody reacts with Src family kinases (Huang and McNamara).

**Huang, Y. Z. and McNamara, J. O.** Mutual regulation of Src family kinases and the neurotrophin receptor TrkB. *J Biol Chem* **285**, 8207-17.
