# Supplementary material for: Interplay between Cell Migration and Neurite Outgrowth Determines SH2B1β-Enhanced Neurite Regeneration of Differentiated PC12 Cells
Source: PLoS One. 2012 Apr 23;7(4):e34999. doi: 10.1371/journal.pone.0034999 (PMC3335126; doi:10.1371/journal.pone.0034999)
Supplement: Figure S5 — Overexpression of SH2B1β increases cell migration. PC12-GFP and PC12-SH2B1β cells were differentiated and subjected to wound healing as described in Figure 1. Live cell images were taken on healing days 0 and 23 using Carl Zeiss Observer Z1 microscope. Scale bar: 200 μm. (DOC) [file pone.0034999.s005.doc]

**
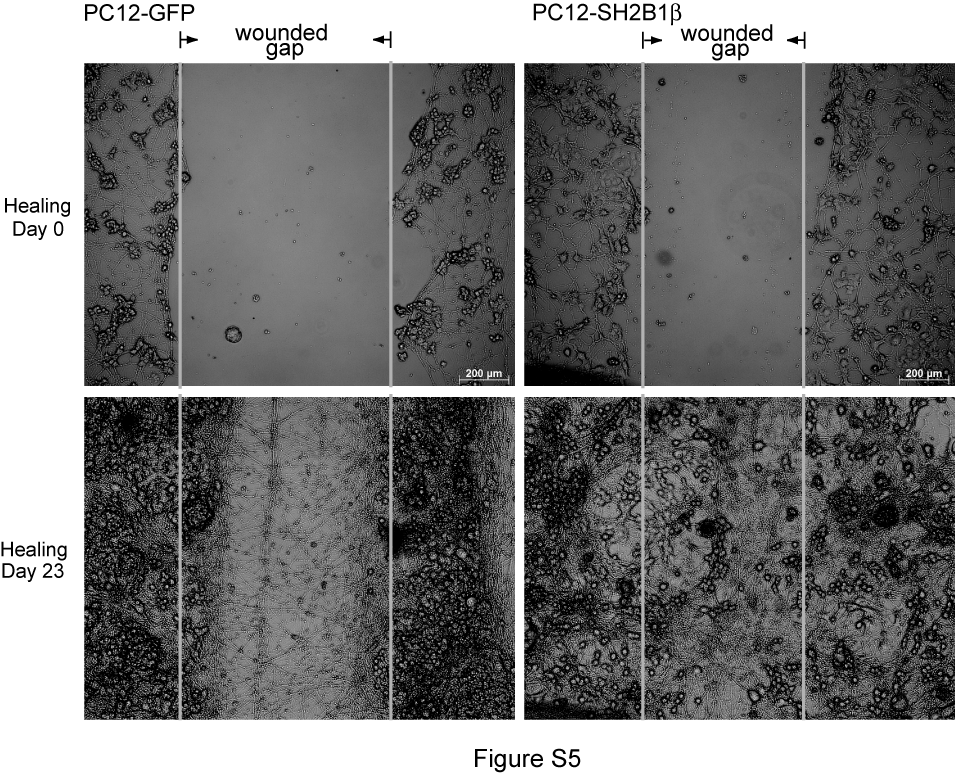
**

**Figure S5 Overexpression of SH2B1β increases cell migration**

PC12-GFP and PC12-SH2B1β cells were differentiated and subjected to wound healing as described in Figure 1. Live cell images were taken on healing days 0 and 23 using Carl Zeiss Observer Z1 microscope. Scale bar: 200 μm.
