# Supplementary material for: Interplay between Cell Migration and Neurite Outgrowth Determines SH2B1β-Enhanced Neurite Regeneration of Differentiated PC12 Cells
Source: PLoS One. 2012 Apr 23;7(4):e34999. doi: 10.1371/journal.pone.0034999 (PMC3335126; doi:10.1371/journal.pone.0034999)
Supplement: Figure S6 — PMA treatment increases SH2B1β levels. PC12-SH2B1β cells were differentiated and subjected to wound healing protocol. On differentiation day 8, un-wounded (U) or wounded cells were treated with or without 162 nM PMA. Cell lysates were collected from un-wounded (U) cells and cells during healing days 0–5. Equal amount of proteins from lysates was resolved via SDS-PAGE and immunoblotted with anti-SH2B1 and anti-HDAC antibodies. Polyclonal antibody to rat SH2B1 was generously provided by Dr. Christin Carter-Su at the University of Michigan, USA. (DOC) [file pone.0034999.s006.doc]

**
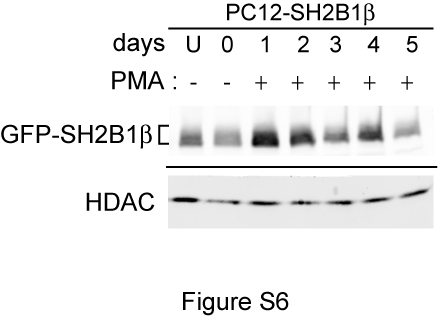
**

**Figure S6 PMA treatment increases SH2B1β levels**

PC12-SH2B1β cells were differentiated and subjected to wound healing protocol. On differentiation day 8, un-wounded (U) or wounded cells were treated with or without 162 nM PMA. Cell lysates were collected from un-wounded (U) cells and cells during healing days 0-5. Equal amount of proteins from lysates was resolved via SDS-PAGE and immunoblotted with anti-SH2B1 and anti-HDAC antibodies.

Polyclonal antibody to rat SH2B1 was generously provided by Dr. Christin Carter-Su at the University of Michigan, USA.
